# Development and Evaluation of Statewide Prospective Spatiotemporal Legionellosis Cluster Surveillance, New Jersey, USA

**DOI:** 10.3201/eid2803.211147

**Published:** 2022-03

**Authors:** Jessie A. Gleason, Kathleen M. Ross

**Affiliations:** New Jersey Department of Health, Trenton, New Jersey, USA

**Keywords:** Legionellosis, Legionnaires’ disease, surveillance, bacteria, water-borne diseases, New Jersey, United States

## Abstract

Incidence of Legionnaires’ disease is increasing, particularly in the Mid-Atlantic states in the United States; since 2015, New Jersey has documented ≈250–350 legionellosis cases per year. We used SaTScan software to develop a semiautomated surveillance tool for prospectively detecting legionellosis clusters in New Jersey. We varied temporal window size and baseline period to evaluate optimal parameter selections. The surveillance system detected 3 community clusters of Legionnaires’ disease that were subsequently investigated. Other, smaller clusters were detected, but standard epidemiologic data did not identify common sources or new cases. The semiautomated processing is straightforward and replicable in other jurisdictions, likely by persons with even basic programming skills.

Legionellosis, a bacterial disease caused by *Legionella*, can manifest as either Legionnaires’ disease or Pontiac fever. Legionnaires’ disease causes severe pneumonia, often requiring hospitalization, and has a fatality rate of 10%–25%, whereas Pontiac fever is generally milder and resolves on its own. In extremely rare cases, *Legionella* can cause extrapulmonary infections, such as endocarditis or wound infections. 

*Legionella* bacteria are found naturally in freshwater environments, such as lakes and streams, but if the bacteria enter human-made water systems with conditions favorable to growth, such as hot tubs, cooling towers, and plumbing systems, *Legionella* can become a health concern. People develop Legionnaires’ disease or Pontiac fever primarily by inhaling aerosolized water droplets containing the bacteria. Any source of aerosolized water is a potential mode of transmission: shower heads, faucets, hot tub jets, decorative fountains, medical devices. Less commonly, aspiration of contaminated drinking water can transmit the bacteria. Extrapulmonary infections result from direct inoculation or secondary hematogenous spread from the lung. Since 2000, the incidence of Legionnaires’ disease has been on the rise, particularly in the Mid-Atlantic states in the United States (*1*).

In accordance with communicable disease reporting regulations detailed in the New Jersey Administrative Code, healthcare providers must report diagnosed cases of legionellosis within 24 hours of laboratory confirmation to the health department local to where the case-patient resides. Local health departments are responsible for investigating all cases of legionellosis occurring within their jurisdictions that are reported to the New Jersey Communicable Disease Reporting and Surveillance System (CDRSS). Investigations include interviewing each case-patient using a standardized questionnaire to gather additional information about possible exposures to *Legionella* during the incubation period, such as spending a night away from home, visiting a healthcare facility, or being near a hot tub. These data are used to identify epidemiologic links between cases and determine the need for outbreak investigations, which are critical for detecting transmission sources and implementing control measures. These outbreaks, or clusters, are easily identified when ≥2 persons with diagnosed legionellosis report the same exposure location during their incubation periods within a 12-month period. Since 2015, New Jersey has documented ≈250–350 legionellosis cases per year. However, outbreaks account for <10% of reported legionellosis cases in New Jersey; remaining cases are classified as sporadic occurrences, defined as isolated events with no known epidemiologic link to other reported cases or confirmed outbreak sources.

Sporadic cases might share a common source of exposure in the community, such as cooling towers (*2*,*3*), underlying issues with a water utility (*4*,*5*), decorative fountains (*6*), or a wide variety of other sources (*3*). Despite exhaustively reviewing epidemiologic, environmental, and microbiological information collected during case investigations, identifying common sources or even linking multiple cases with spatiotemporal associations can be difficult (*7*). Without a reported common source location, such as a specific building, or a sudden unexplained increase in reported cases, clusters might go undetected because cases manifested in an unusual geographic or temporal pattern that may be caused, for example, by an intermittently operated cooling tower or because disease baselines are very high (*8*,*9*). 

In addition to using standard surveillance practices, some jurisdictions have developed systems to enhance prospective detection of legionellosis clusters using SaTScan software (https://www.satscan.org). SaTScan is a free software program that can be used to identify disease clusters across both space and time by calculating a space-time scan statistic for every possible combination of geographic extent and length of time within specified ranges. The test statistics indicate an unusual disease cluster if the number of observed cases within each spatiotemporal window exceeds the number of expected cases (*10*,*11*). SaTScan users have to specify parameter settings to determine which clusters will be detected (*12*). Local health departments, such as the New York City (NYC) Department of Health and Mental Hygiene (DOHMH) (*10*,*13*) and the Allegheny County (Pennsylvania) Health Department (*9*), have developed automated programs to run SaTScan to detect legionellosis clusters in real time. NYC DOHMH’s automated prospective cluster detection system detected a large outbreak of legionellosis associated with a cooling tower before it was identified using traditional methods (*9*,*13*).

Given the challenges in finding epidemiologic links and spatiotemporal associations among cases, we used SaTScan to develop and evaluate a semiautomated system that was successful in prospectively identifying active clusters. Here, we document the methods used to create the system and provide a technical guide and a description of the detected clusters (Tables 1, 2; Appendix).

**Table 1:** Summary of results for different parameter file selections across 3 detected and investigated community clusters, New Jersey, USA, 2019*

Cluster	County	Duration of SaTScan signal	Cluster radius size, km	Cases, no.	First RI/max RI
1	Mercer	2 weeks	2.78	3	130 d/175 d
2	Union†	6–13 weeks	6.77–6.96	8–22	184 d/8,130 y
3	Morris	4 weeks	4.59	7	3.3 y

*RI, recurrence interval. †Because this outbreak was prolonged and geographically large and results varied based on parameter files and timing of the scan, ranges are indicated.

**Table 2:** Comparison of results based on parameter selection using performance measures for legionellosis clusters prospectively detected with recurrence intervals ≥100 d, New Jersey, USA, 2019*

Cluster	County	Performance measure	5-y baseline			2-y baseline	
			90-d window, parameter 1	30-d window, parameter 2		90-d window, parameter 3	30-d window, parameter 4
1	Mercer	Detected earliest		✓†			
Longest RI		✓			
2	Union	Detected earliest	✓	✓		✓	✓†
		Longest RI	✓				
3	Morris	Detected earliest	✓				
		Longest RI	✓				

*Check marks (✓) indicate parameter files that satisfied the performance measure; blank cells indicate parameter files that did not satisfy performance measure. RI, recurrence interval. †Smaller cluster of cases was detected 2 weeks before the other scans detected it with a RI of 184 d. RI was ≥100 d in the next weekly scan but was detected in the following weekly scan.

## Methods

### Data Sources

SaTScan scans require a case file and coordinate file. The case file includes 1 record for each case, including their geocoded census tracts and event dates (earliest among date of illness onset, specimen collection, or report). The coordinates file includes geographic coordinates for the centroid of each census tract, identified by a unique location identification. Census blocks, counties, postal codes, or geographic units can alternatively be used as the geographic unit for coordinate files. The US Census Bureau provides geographic state census tract layers, which can be projected and displayed in ArcGIS (https://www.arcgis.com). 

### SaTScan Parameter Selections

SaTScan requires users to select parameters for analyses being conducted. The SaTScan user guide provides guidance on selecting parameter files (*14*). In brief, we created 4 parameter files using the SaTScan user interface and saved them in .prm format files to be used for weekly analyses. Parameter files locate the case and coordinates file names and file paths. We selected the prospective analysis and the space-time permutation model options. Prospective analysis is used to detect disease outbreaks early when analyses are conducted on a routine basis (e.g., daily, weekly). We searched only for alive clusters, defined as active clusters that must reach the study period end date.

For prospective analyses, SaTScan users can adjust analysis parameters, including the duration of study period baselines, temporal windows, time aggregation, and maximum spatial cluster sizes to optimize detecting clusters. Given the potential effects of parameter selections on results, we evaluated 4 different combinations of analysis parameters. Because of increasing legionellosis incidence in New Jersey, we conducted analyses using both 2- and 5-year study periods to establish a stable baseline. To adjust the length of baseline periods, users can define start dates. We further adjusted the temporal window size to account for clusters of varying time lengths. We conducted analyses using both 30-day (acute) and 90-day (prolonged) window sizes. We used the default maximum spatial cluster size of 50% for all analyses, to enable detection of both small and large clusters.

Finally, spatiotemporal analyses can be very computer intensive. To reduce computing time, case data can be aggregated into time intervals. For all 4 of our parameter files, we chose to aggregate data into 7-day windows to reduce data size and ease processing time. Early test runs of the different parameter files found these settings resulted in both reasonable processing times and sensitivity for detecting clusters. 

### Automation Process

To automate the process (Appendix), we prepared case files in SAS version 9.4 (*15*). We exported legionellosis data from CDRSS using SAS/ACCESS Interface to Oracle and used the SAS GEOCODE procedure to assign each case to a census tract of residence using a street address. We then exported a case file and used it to replace the previous week’s case file of exactly the same file name. The SAS program calls open a command prompt window and points to the directory where SaTScan is located and launches it in batch mode with the 4 parameter files. Weekly analyses require that the start and end dates be changed each week relative to the current date. Although these dates can be adjusted manually in the SaTScan interface on a weekly basis, we automated this process by defining the new dates on the command prompt which overrides the start and end dates specified in the parameter file. After SaTScan completes scanning for clusters, it creates results files and saves them in standard text-based format to a file path defined within the parameter file. The SAS program generates and sends emails to users with results files attached for review.

### Signal Detection and Public Health Response

Results files contain information about the detected clusters, including the location and size of the cluster, number of cases, expected number of cases, p values, and recurrence intervals. We evaluated all clusters with a recurrence interval ≥100 days, the equivalent of 1 expected false positive every 100 days, the value used by the NYC daily prospective cluster detection system (*13*). Recurrence intervals, a reciprocal of p values, are a measure of how often an observed cluster would be of that size or larger by chance (*14*). Public health departments can use recurrence intervals to minimize the number of false signals generated during a selected time period (*11*).

When a cluster with a recurrence interval ≥100 days was identified, disease investigators closely reviewed the cluster results (cluster radius, recurrence interval, and number of cases). Some clusters with short recurrence intervals (e.g., <365 days), small numbers of cases (e.g., 2 or 3), or large radii were closely monitored in subsequent weekly analyses to evaluate any changes to the cluster and other case details. Other cluster signals with longer recurrence intervals, larger numbers of cases, or smaller geographic radii spurred investigators to take immediate additional action, including reviewing details from each case investigation to determine any common exposures. If no common exposures were identified, case-patients associated with the suspected cluster were reinterviewed using the New Jersey cluster hypothesis generating questionnaire. Based on information gathered from these interviews, we considered whether further investigation and an environmental assessment were needed.

If the investigation confirmed a likely outbreak source, we removed the cases associated with the cluster from future analyses, at a time decided on a case-by-case basis; however, a general guideline was 4 weeks, roughly 2 incubation periods, after the cluster was no longer statistically significant. The cases were removed from the case file to ensure future clusters in the same location would not be missed.

## Results

We ran the SAS/SaTScan program for each week of 2019, starting January 1, and identified 3 clusters. We compared the 4 weekly results files created to assess how differences in the analysis parameters selected affected the detection of signals.

### Cluster 1

The first analysis on January 1, 2019 detected a cluster with 3 cases and a recurrence interval of 130 days in Mercer County. The following week, the cluster’s recurrence interval increased to 175 days (cluster 1, Table 1). Public health officials interviewed case-patients associated with the cluster but identified no common exposure among the case-patients or additional cases in the following weeks. Because of the short recurrence interval, this signal possibly represented a false positive.

### Cluster 2

An analysis performed on April 17, 2019, detected a cluster of 10 cases in Union County with a recurrence interval as long as 15 years, depending on the parameter file. New Jersey Department of Health (NJDOH) requested that local health departments reinterview the case-patients using the New Jersey cluster hypothesis generating questionnaire. The interviews identified no common sources of exposure, but additional cases associated with the cluster continued to be reported. At its peak on May 15, 2019, the recurrence interval increased to 8,130 years. Ongoing weekly SaTScan analyses identified additional clusters, which were further investigated to determine whether they were part of the larger, primary cluster. 

Investigators at NJDOH were able to present the SaTScan results to public health management as evidence that there was an ongoing, unexplained statistically significant increase in disease above the baseline that warranted additional public health resources. Subsequently, NJDOH requested Epi-Aid rapid epidemiologic assistance from the Centers for Disease Control and Prevention to help guide an epidemiologic and environmental investigation to determine the extent of disease and identify and mitigate any risks of continued exposure. 

We identified 21 cases with illness onset dates during March 8–13, 2019; median patient age was 72 years (range 46–95 years). All patients were hospitalized, and 5 died. The investigative team identified several outdoor aerosol-generating devices determined to be conducive for *Legionella* growth. Devices identified as at-risk were required to undergo remediation to eliminate the risk of *Legionella* growth and transmission. Although we identified no definitive links, no additional cases were reported after the conclusion of the Epi-Aid.

This cluster investigation and its findings were unique. The cases occurred over a span of 11 weeks, with 0–3 cases occurring per week. The case-patients resided across 15 different municipalities, many with their own local health department. Local health departments only have access to reports of disease occurring in their jurisdiction and are therefore not aware of cases occurring in neighboring jurisdictions. SaTScan was useful for linking cases in space and time across several jurisdictions.

### Cluster 3

An analysis performed on June 26, 2019, detected a cluster of 7 cases with a recurrence interval of 3.3 years in Morris County. The initial interview of the case-patients did not identify a common source of exposure. In response to the suspected cluster, case-patients were reinterviewed using the New Jersey cluster hypothesis generating questionnaire. The investigation identified 6 case-patients with illness onset during April 28–June 25, 2019, all of whom reported visiting the same hardware store during their incubation periods.

Investigators visited the hardware store to identify any potential sources of aerosolized water and discovered that a filled hot tub had been on display and operating from January through June 22, 2019. Hot tubs not appropriately treated and maintained can become contaminated with *Legionella* and are a known source of outbreaks. During the visit, investigators identified notable concerns about the operation of the hot tub including no written records to indicate what test parameters were being measured, no implementation of a draining or cleaning schedule, no written maintenance protocols, and no clear understanding by staff of the potential risk of *Legionella* growth. 

### Signal Detection and Parameter Comparison

Different parameter selections produced different results. Two performance measures, early detection and length of recurrence intervals, were compared across the 4 analysis parameter combinations (Table 2). Whereas shorter 30-day maximum temporal windows (which we used for parameter files 2 and 4) detected clusters earlier, longer 90-day windows captured more cases with longer recurrence intervals. For prolonged clusters, using the longer maximum temporal windows maintained statistical significance in subsequent weekly scans. In the Union County and Morris County clusters, cases occurred over 30-day periods, suggesting ongoing but intermittent sources of exposure, likely better detected using longer temporal windows and longer baseline parameters.

## Discussion

We identified 3 suspected community clusters of Legionnaires’ disease in New Jersey using a semiautomated prospective cluster detection surveillance tool developed using SaTScan software. Although public health departments would possibly have detected the 3 community clusters using standard surveillance practices, they might not have been alerted to them as soon or been as promptly reactive without the strong recurrence interval signals from SaTScan. SaTScan also identified additional potential cases in clusters that were not associated by public health investigators alone. Cluster detection validated disease investigators’ suspicions of a possible increase in cases in space and time and provided additional statistical support for taking resource-intensive action.

Users should select parameters on the basis of their jurisdiction’s needs because those choices can meaningfully vary results (*16*). No one model will be most effective for the surveillance needs of all urban or rural, or city, county, or state jurisdictions, so investigators would benefit from piloting and exploring a variety of different options and performing continued surveillance using different parameters, baseline periods, and geographic units. NJDOH will continue to use the results from the 4 different sets of SaTScan parameters to identify possible disease clusters because they have different abilities in different contexts. It is notable that SaTScan does not adjust the recurrence interval when making multiple comparisons running different sets of SaTScan parameters simultaneously.

Allegheny County demonstrated the ability of the modified NYC program to detect simulated outbreaks except when fewer cases occurred over a longer timeframe (*9*). The slowly occurring outbreak that Allegheny County simulated was based on information from a published description of a suspected outbreak in New Jersey associated with an area of a community water system, confirmed through retrospective cluster surveillance using SaTScan (*4*). This outbreak was thought to occur over 5 years, not 163 days as simulated. Allegheny County used 1- and 2-year baseline periods, possibly not long enough to detect the cluster. Results from New Jersey when using a 5-year baseline demonstrate the ability of scans with longer baselines to detect clusters with longer recurrence interval signals, suggesting that longer baselines should be considered more often. However, using baselines >1 year long increases the risk for population shift bias, which occurs when the background population increases or decreases faster in some areas than in others, which in turn can produce biased p values. 

A multistate analysis of data from SaTScan scans to detect prospective clusters missed certain cluster types, such as travel-associated clusters or those with prolonged times between cases (*8*). SaTScan is not likely to improve detection of small clusters (e.g., <2 cases associated with a single facility) (*8*); however, current public health surveillance methods sufficiently detect these most common types. Incorporating SaTScan-generated prospective cluster analyses as part of the New Jersey Legionnaires’ disease surveillance system has enabled us to identify geographically larger clusters crossing multiple local health jurisdictions that usually require additional public health surveillance tools to verify. 

Our outbreak case removal practice differed from the NYC cluster surveillance system, which removes all cases identified during the cluster period from the baseline, regardless of evidence linking them to the cluster (*13*). Although the population of NYC is similar to that of the entire state of New Jersey, the geographic coverage area is much smaller. Removing all cases statewide, even those clearly not associated with a cluster during an outbreak period, might restrict our ability to detect future prolonged clusters in other locations. However, removing all cases in an outbreak area might inadvertently remove cases unrelated to the outbreak and artificially lower the true baseline of disease, which could lead to false cluster detection in future analyses.

Legionnaires' disease diagnosis in the United States relies largely on the *Legionella* urinary antigen test (UAT), which provides rapid results for diagnosing Legionnaires’ disease. However, UATs only identify infections caused by *L. pneumophila* serogroup 1, and because other *Legionella* spp. are also pathogenic, public health surveillance systems may be underdiagnosing and underreporting cases (*17*). Healthcare providers, concurrent with UAT testing of a patient, should consider collecting a respiratory specimen for *Legionella* culture or PCR tests that can identify other *Legionella* species and serogroups. 

Some jurisdictions may find it practical to adapt an existing system for their surveillance needs. NYC DOHMH created a SAS program to automate daily spatiotemporal cluster detection for reportable communicable diseases (*13*), which Allegheny County modified for use in its own jurisdiction (*9*). New Jersey has a population of just under 9 million and comprises 21 counties of varying population densities—most largely urban, but some rural. Our statewide setting could provide a template to assist other states, regions, and countries in developing their own tools to prospectively detect spatiotemporal legionellosis clusters across multiple jurisdictions. The semiautomated process developed in New Jersey (Appendix) may similarly be replicable for other jurisdictions, even by basic SAS users, without the need to include macros.

In conclusion, our prospective cluster detection system identified 3 community outbreaks of Legionnaires’ disease that led to public health investigations. Prospective cluster detection can be used in conjunction with standard epidemiologic methods, which are successful at identifying environmental sources such as premise plumbing in a single facility. Using the strategies together has provided better public health response in New Jersey.

Appendix Technical guide for SaTScan program for prospectively detecting disease clusters.
