# Supplementary material for: Development and Evaluation of Statewide Prospective Spatiotemporal Legionellosis Cluster Surveillance, New Jersey, USA
Source: Emerg Infect Dis. 2022 Mar;28(3):625–30. doi: 10.3201/eid2803.211147 (PMC8888220; doi:10.3201/eid2803.211147)
Supplement: Appendix — Technical guide for SaTScan program for prospectively detecting disease clusters. [file 21-1147-Techapp-s1.pdf]

Article DOI: https://doi.org/10.3201/eid2803.211147

Development and Evaluation of Statewide Prospective Spatiotemporal Legionellosis Cluster Surveillance, New Jersey, USA

Appendix.

Part I: Create Your SaTScan Coordinates File

SaTScan requires a coordinates file which provides the geographic coordinates for each location ID. Each row represents one geographical location. In NJ, we use census tracts as our geographical unit. Our coordinates file includes census tract number (identified by the GEOID – see page X for more information) and the X-Y coordinates of the census tract centroid. We used ArcGIS to produce the X-Y coordinates of each census tract centroid.
TIP: You only need to create the coordinates CSV file one time. Save to a folder on your computer to be input into SaTScan later.

Part II: Create Your SaTScan Case File

The case file provides information about the cases, and it is used for all probability models. Each row in the case file represents one or more cases of disease for each location (census tract) and event date (the illness onset date if available; otherwise the specimen collect date, or if also missing, the report date).
The case file must be regularly updated to include the most recently reported cases. We generate an updated case file each time prior to running SaTScan. To prepare our case file, we use SAS 9.4. Briefly, this includes:

1. **Import data**. Legionellosis data is imported from our communicable disease database by using SAS/ACCESS Interface to Oracle, which includes SAS Data Connector to Oracle. Data variables include street address, city, state, zip code, illness onset date, specimen collection date, and report date.
2. **Format case data**. SaTScan requires that the variables in the case file are formatted in a specific way. For space-time permutation models, the case file requires, at minimum, the following variables fields: geocoded location ID, event date, and the number of cases observed.
3. **Geocode cases**. We assign the census tract centroid to each case residential address.
4. **Export case file**. Save the case file to your computer.

Part III: Create Your SaTScan Parameter File(s)

Using the SaTScan interface, select the analysis parameters and file pathway locations for Coordinates and Case files for the analysis being conducted as well as the folder location and name of Results file. Refer to the SaTScan User Guide for further explanation regarding parameter selection. Your parameter selections are saved with a ".prm" file extension and stored in an ASCII text file format.

Tip: Given the potential impact of parameter selection on cluster detection, our analysis uses several different combinations of analysis parameters, resulting in the creation of four different parameter files. Please refer to the paper for further justification.

```
LIBNAME X "insert folder location pathway";

LIBNAME MYLIB2 ORACLE USER="insert" PASSWORD="insert" PATH="(DESCRIPTION=
            (ADDRESS=
                (PROTOCOL=insert)
                (HOST=insert)
                (PORT=insert)
            )
            (CONNECT_DATA=
                (SERVER=insert)
                (SID=insert)
             )
                )" ;
RUN;

DATA X.LEGIONELLOSIS;
        SET
```

Reading in case data from NJ's Communicable Disease Database. Our case data is managed in Oracle, a type of a relational database management system (DBMS). You can use a SAS/ACCESS LIBNAME statement to connect the SAS Interface to a DBMS.
TIP: Work with your IT Department to determine how your data is stored/managed and to identify connection options you may need. Alternatively, consider asking your IT Department to set up a Microsoft Access database that is updated in real-time (or near real-time). You can easily read in the data from Access into SAS.

```
MYLIB2.DLY_LEGI_CLSTER_FORLAST360 ;

        LENGTH CASENUM 8. ;

        ILLNESS_ONSET = DATEPART(ILLNESS_ONSET_DATE);
        PUT ILLNESS_ONSET MMDDYYD10. ;
        FORMAT ILLNESS_ONSET MMDDYYD10. ;

        SPECIMEN_DATE =
        DATEPART(MIN_SPECIMEN_COLLECTED_ON);
        PUT SPECIMEN_DATE MMDDYYD10. ;
        FORMAT SPECIMEN_DATE MMDDYYD10.

        DATE_FOR_REPORT = DATEPART(DATE_FOR_REPORT);
        PUT DATE_FOR_REPORT MMDDYYD10. ;
        FORMAT DATE_FOR_REPORT MMDDYYD10. ;


SATSCANDATE=ILLNESS_ONSET;
IF SATSCANDATE=. THEN SATSCANDATE=SPECIMEN_DATE;
IF SATSCANDATE=. THEN SATSCANDATE=DATE_FOR_REPORT;
FORMAT SATSCANDATE MMDDYYD10.;
```

Preparing Case Data File:
First, create and format a SaTScan date variable. If available, the illness onset date is used as the event date. For cases missing illness onset date, the collection date of the clinical specimen is used and when this is also missing the case report date is selected.
TIP: SaTScan requires that date is entered in a specific format. We recommend the SAS date format MMDDYY10.

```
CASENUM=1;
```

Must identify number of case observation per location ID (e.g., geographical unit, such as a census tract).
TIP: Alternatively, identify CASENUM=1 to identify each case is equal to one observation and SaTScan will calculate number of cases per location ID.

```
STREET_=STREET;
CITY_=CITY;
STATE_=STATE;
ZIP_=ZIP*1;

RUN;
```

Renaming our address variables to match the PROC GEOCODE defaults (used later).

```
DATA X.LEGIONELLOSIS2;
        SET X.LEGIONELLOSIS;

IF CASE_STATUS IN ('CONFIRMED',
'REPORT UNDER INVESTIGATION
(RUI)',
'POSSIBLE', 'PROBABLE');
IF REPORT_STATUS NE 'MERGED';
```

Define case inclusion and exclusion criteria. In the analyses we include confirmed cases and reports under investigation and exclude duplicate reports (status=merged).

```
/*OUTBREAK #1*/
IF CASE_ID IN (Insert Case-ID values separated by commas)
THEN DELETE;

/*OUTBREAK #2*/
IF CASE_ID IN (Insert Case-ID values separated by commas)
THEN DELETE;

RUN;
```

TIP: Remove cases associated with previously identified outbreaks – justification provided in paper.

Now the case file data is ready to be geocoded. We used the SAS GEOCODE procedure to find the census tract of each cases' home street address. PROC GEOCODE uses United States Census TIGER data for street geocoding (LOOKUP files). These LOOKUP files must be downloaded to your computer first. Below we describe the steps of this process:

1. Download prebuilt U.S. street lookup data from the SAS Maps online website and unzip the file.
    a. The download includes a ZIP file with ten CSV files, a read me file, and a SAS program named ImportCSVfiles.sas program.
    b. The ImportCSVfiles.sas program is used to create the street lookup data for PROC GEOCODE
2. Open ImportCSVfiles.sas program in SAS and set PATHIN and PATHOUT to directories you want, but make sure that you create the directory specified in PATHOUT before running ImportCSVfiles.sas.

```
%let PATHIN='insert folder location;       /* Directory with files from the zip archive */
%let PATHOUT='insert folder location\Data; /* Location to write geocoding data sets */
```

3. Use the information from the ReadMe.txt file for these values:

```
%let source= US Census Bureau TIGER/Line files; /* Original source for the lookup data */
%let release=2018;  /* Year original data published  */
```

4. Run the SAS program with your settings. After the program finishes, a library named Lookup is ready to geocode street addresses.

You can now run PROC GEOCODE!

TIP #1: Running the SAS program to process the CSV LOOKUP files is slow, but it only needs to be run once on each PC.

```
LIBNAME LOOKUP2 'insert folder location pathway for LOOKUP files\StreetLookupData_94-2017SASv9.4\Data';

PROC GEOCODE
METHOD=STREET
ADDRESSVAR = STREET_
ADDRESSCITYVAR=CITY_
ADDRESSZIPVAR=ZIP_
ADDRESSSTATEVAR=STATE_

DATA = X.LEGIONELLOSIS2 /*this is your case file*/

OUT = X.GEOCODED_CASE /*your new geocoded file*/

LOOKUPSTREET=LOOKUP2.usm /*made the folder name lookup*/
ATTRIBUTE_VAR = (BLOCK, TRACT); /*renamed this to match name in the uss file*/
RUN;
```

TIP: Once the LOOKUP files are read in, you now run PROC GEOCODE with your case address data. You can also specify an output data set, the geocoding method, and additional attributes variables to associate with the matched addresses.

```
/*COUNTY NAME NEEDS TO BE CONVERTED TO NUMBER CODE TO CREATE THE GEOID10*/
DATA COUNTYCODE;
        INFILE DATALINES DSD;
        INPUT  @1 COUNTY $10.
               @13 COUNTYNUM $3.;


DATALINES; .
ATLANTIC      001
BERGEN        003
BURLINGTON    005
CAMDEN        007
CAPE MAY      009
CUMBERLAND    011
ESSEX         013
GLOUCESTER    015
HUDSON        017
HUNTERDON     019
MERCER        021
MIDDLESEX     023
MONMOUTH      025
MORRIS        027
OCEAN         029
PASSAIC       031
SALEM         033
SOMERSET      035
SUSSEX        037
UNION         039
WARREN        041
;
RUN;
```

Create dataset with U.S. census county codes.

TIP: The geocoded case file includes census block and tract. However, the complete U.S. Census GEOID is a 11-digit number [2 digit state code + 3 digit county code + 6 digit census tract code].

```
PROC SORT DATA=X.GEOCODED_CASE; BY COUNTY; RUN;
PROC SORT DATA=COUNTYCODE; BY COUNTY; RUN;
DATA GEOCODED_CASE;
MERGE X.GEOCODED_CASE (IN=A) COUNTYCODE (IN=B);
BY COUNTY;
IF A;
RUN;
```

Merge together county code dataset with the geocoded case file.

```
DATA GEOCODE;
        SET GEOCODED_CASE;
LENGTH GEOID10 $11. TRACT2 $6.;
STATENUM=34; /*this is the U.S. census state number*/
/*ADDING LEADING ZEROS*/
TRACT2=TRACT;
IF TRACT2=. THEN DELETE;
TRACTNEW=TRANSLATE(RIGHT(TRACT2),'0','');

GEOID10=COMPRESS(STATENUM||COUNTYNUM||TRACTNEW);

IF STATE NE 'NJ' THEN DELETE;

RUN;
```

This data step identifies NJ's state census code and the rest formats the state, county, census and blocks codes into the GEOID10 which is assigned to each census tract.

This removes all non–New Jersey cases.

```
PROC SQL;
	CREATE TABLE GEOCODE_CASE AS
	SELECT GEOID10, CASENUM, SATSCANDATE,
	CASE_ID
		FROM GEOCODE;
RUN;
```

Additional file formatting to clean file and include only necessary variables, in proper order to be consistently read into SaTScan.

```
PROC EXPORT DATA=GEOCODE_CASE
	OUTFILE="insert folder location pathway\CASE.TXT"
DBMS=DLM REPLACE;
PUTNAMES=NO;
RUN;
```

This final case file is output to your designated folder location.

Data Quality Check

The following code merges the original case file with the geocoded file to identify cases that were not geocoded. A data file is output with all non-geocoded cases. After every weekly SaTScan run this file is output and program staff review to find cases that could not be geocoded. If it is a data entry error these are corrected, and the code is re-run.

```
DATA MAIN1; SET X.LEGIONELLOSIS2; TEST1=1; RUN;
DATA GEOCHECK; SET GEOCODE; TEST2=2; RUN;

PROC SORT DATA=MAIN1; BY CASE_ID; RUN;
PROC SORT DATA=GEOCHECK; BY CASE_ID; RUN;
DATA QCCHECK;
	MERGE MAIN1 GEOCHECK;
	BY CASE_ID;
RUN;

DATA QCCHECK2;
	SET QCCHECK;
KEEP CASE_ID STREET CITY ZIP DATE_FOR_REPORT;
TEST3=TEST2-TEST1;
IF TEST3 NE 1;
RUN;

PROC EXPORT DATA=QCCHECK2
OUTFILE=' insert folder location pathway '
DBMS=CSV
REPLACE;
RUN;
/******************************************/
```

TIP: This is a data quality check. A data file is output with all non-geocoded cases. After every weekly SaTScan run this file is output and program staff review to find cases that could not be geocoded. If it is a data entry error these are corrected, and the code is rerun.

Running SaTScan in Batch Mode

1. Specify folder location where SaTScanBatch.exe file is saved (e.g. c:/Program Files/SaTScan)
2. Create Today's Date [a SAS variable which gives the current date].
3. Define start and end dates to dynamically update SaTScan parameter files
4. Run SaTScan parameter files in batch mode through SAS

```
LIBNAME SATSCAN "C:\Program Files (x86)\SaTScan";
        %LET satscandir = %sysfunc(pathname(satscan));
        %put &satscandir;

/*x opens the command window*/
options noxwait;
x "cd &satscandir"; /*calls open the command window and
points to our directory*/
```

Opens the command window and points to directory where SaTScan batch mode file is located.

```
%let todaysDate = %sysfunc(today(), YYMMDDs10.);
%put &todaysDate;
```

Define start and end dates to dynamically update parameters files that have been created and saved via the SaTScan user interface.

```
/*define start date for first two analyses with 5-year (1826 day)
baseline*/
%let startDate1= %sysfunc(intnx(day,%sysfunc(today()),-1826),YYMMDDs10.);
%put &startdate1;

/*define start date for second two analyses with 2-year (730 day) baseline*/
%let startDate2= %sysfunc(intnx(day,%sysfunc(today()),-730),YYMMDDs10.);
%put &startdate2;

/* Run SaTScan in batch mode*/

/*#1 Parameter File*/
x CMD /c "SaTScanBatch.exe parameter_V1.prm --
enddate=&todaysdate --startdate=&startDate1";

/*#2 Parameter File*/
x CMD /c "SaTScanBatch.exe parameter_V2.prm --
enddate=&todaysdate --startdate=&startDate1";
```

Run SaTScan using parameter files that have been previously created and saved through the SaTScan user interface. In this command prompt we overwrite the dates.

1. Define enddate as today's date
2. Define startdate as startdate macro variables created in step above.

```
/*#3 Parameter File*/
x CMD /c "SaTScanBatch.exe parameter_V3.prm --enddate=&todaysdate --startdate=&startDate2";

/*#4 Parameter File*/
x CMD /c "SaTScanBatch.exe parameter_V4.prm --enddate=&todaysdate --startdate=&startDate2";
```

Email Results Files

After all analyses have completed and results file are output, they are automatically emailed to designated recipients.

TIP: Sometimes, Microsoft Outlook is configured to generate a pop-up asking permission to send the e-mail. This is something you cannot change within SAS. After taking necessary precautions, you can change the Windows registry to bypass the pop-up. Follow the instructions under the Outlook Popup Section (page 4) of Using Mail Functionality in SAS. For more detailed instructions, refer to this Microsoft webpage.

```
*****************************************************************;
filename mymail
email (‘email address 1', ‘email address 2')
subject='RESULTS'
Attach=(
“Insert location where results files are output\Results_1.txt"
"Insert location where results files are output \Results_2.txt"
"Insert location where results files are output \Results_3.txt"
“Insert location where results files are output \Results_4.txt"
);

data _null_;
  file mymail;
  put;
  put 'Cluster Results';
  put;
run;
```

TIP: The results file location and name of file is selected during SaTScan parameter file creation.

TIP: After each run, these files are overwritten so that the name and location remain the same each time.
